# Supplementary material for: Discrimination between Carbapenem-Resistant and Carbapenem-Sensitive Klebsiella pneumoniae Strains through Computational Analysis of Surface-Enhanced Raman Spectra: a Pilot Study
Source: Microbiol Spectr. 2022 Feb 2;10(1):e02409-21. doi: 10.1128/spectrum.02409-21 (PMC8809336; doi:10.1128/spectrum.02409-21)
Supplement: SUPPLEMENTAL FILE 1 — Supplemental material. Download SPECTRUM02409-21_Supp_1_seq9.pdf, PDF file, 5.3 MB [file spectrum02409-21_supp_1_seq9.pdf]

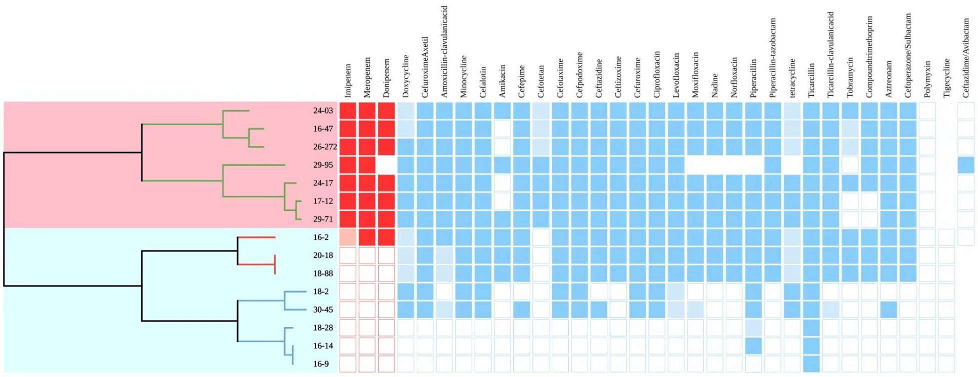

(A)

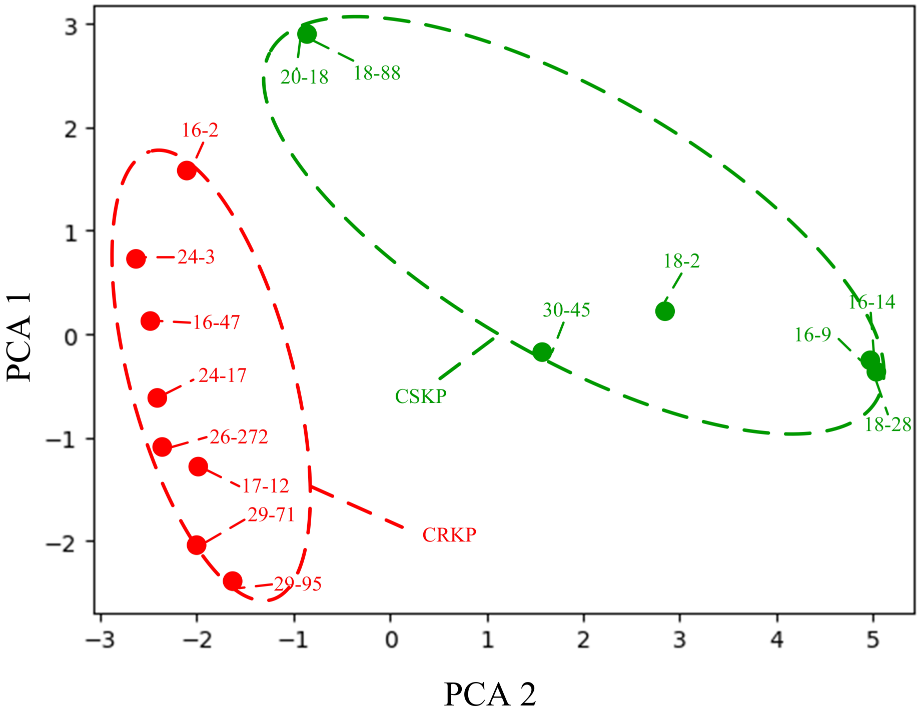

(B)

Supplementary Figure 1 HCA and PCA analyses of antibiotics-resistant profiles in the CSKP and CRKP strains. (A) Clustering analysis of *Klebsiella pneumoniae* strains based on antibiotics-resistant profiles via HCA algorithm. It was noteworthy that sample No. 16-2 was actually CRKP, which was mistakenly classified into the group of CSKP singly based on its antibiotics-resistant phenotype. Red (Carbapenem

resistance) and blue (other antibiotic resistance) squares were used to represent different types of antibiotic resistance: true resistance (filled square), intermediate resistance (semi-transparent square), sensitivity (white square with border), and missing values (white square without border). (B) PCA analysis of *K. pneumoniae* strains based on antibiotics-resistant profiles. CRKP (red dots) and CSKP (green dots) were classified into two separate groups as indicated by the red and green circles, respectively.

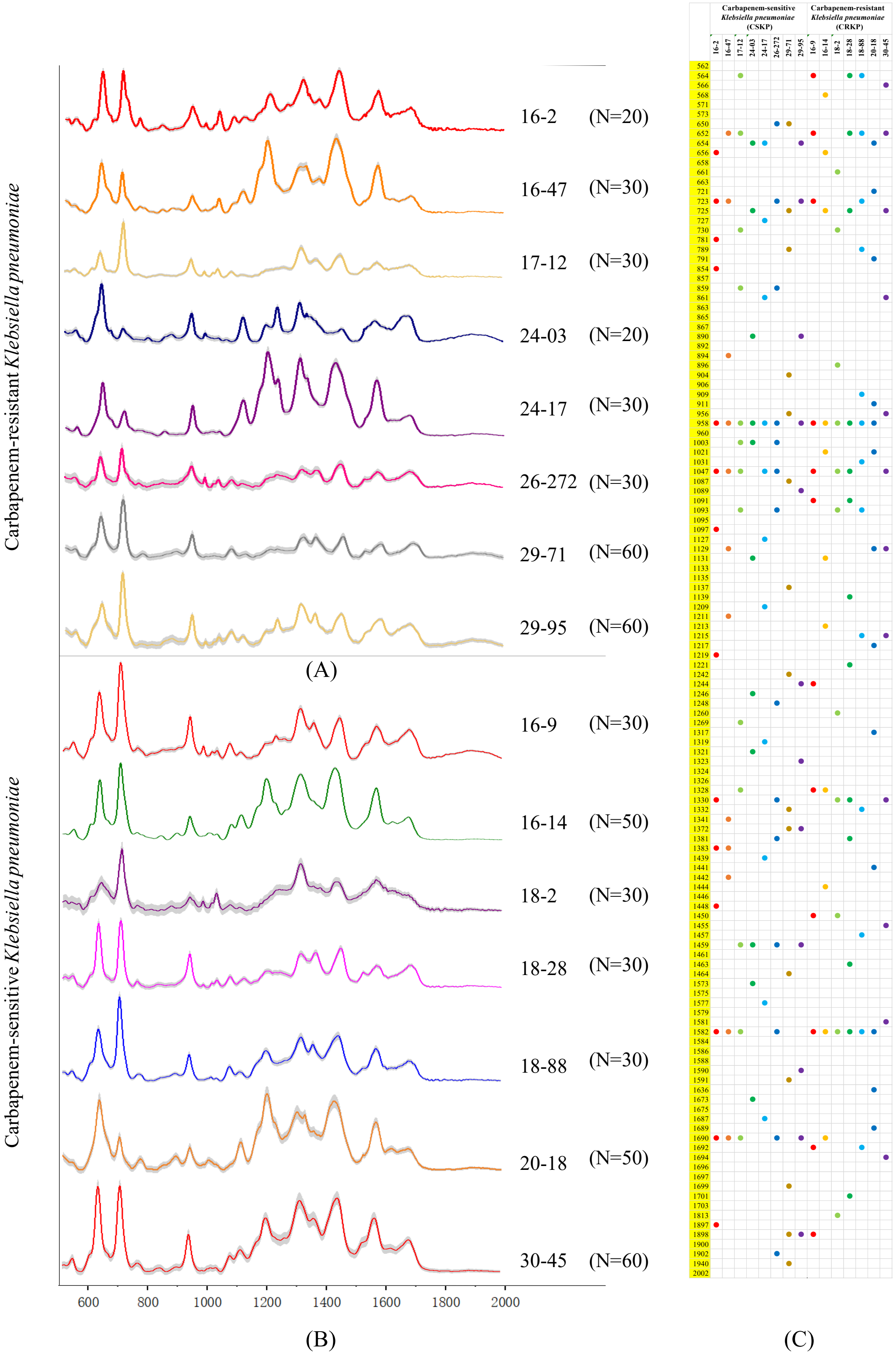

Supplementary Figure 2 Schematic illustration of average SERS spectrum and characteristic peaks for each of *K. pneumoniae* strains. (A) Average SERS spectrum for each of the CRKP strains. (B) Average SERS spectrum for each of the CSKP strains. (C) Distribution of characteristic peaks for each of *K. pneumoniae* strains in dot matrix

plot. N denotes the number of Raman spectra used for generating average Raman spectrum of each *K. pneumoniae* strain.

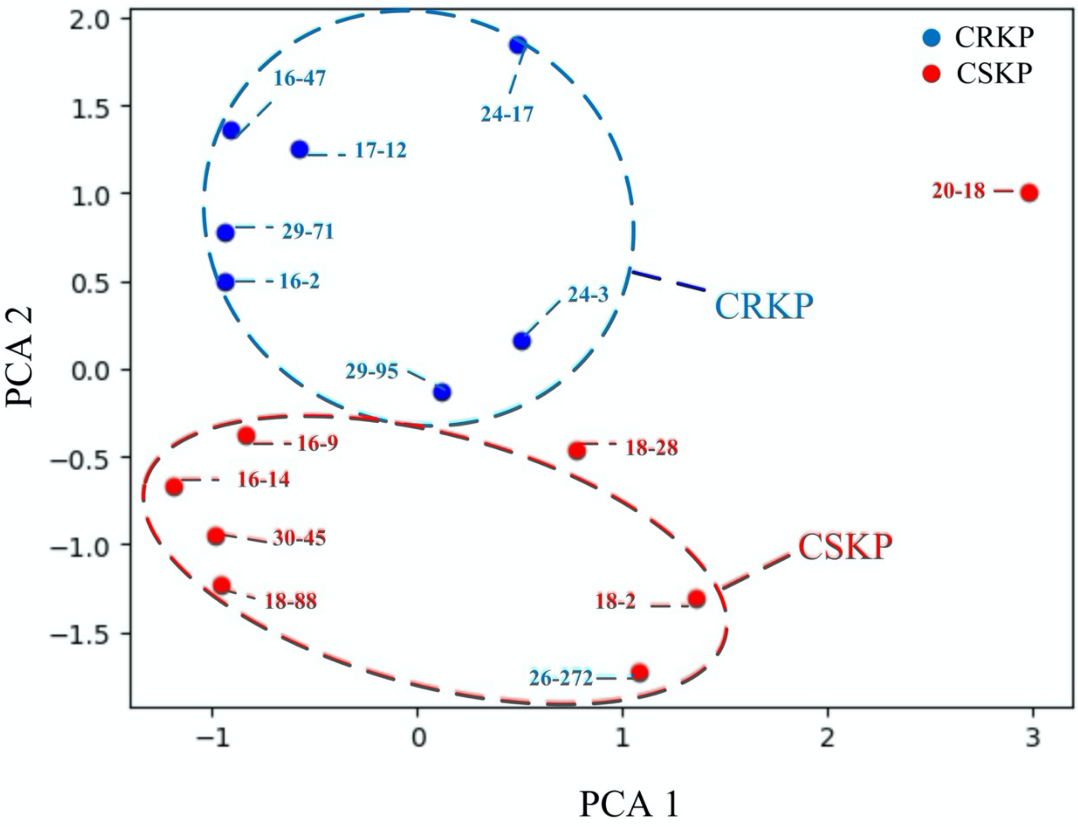

Supplementary Figure 3 Schematic illustration of PCA analysis of characteristics peaks for CSKP and CRKP strains. All the CSKP samples were present in red dots while CRKP samples were present in blue dots. However, it was noteworthy that sample No. 26-272 was CRKP but mistakenly identified as CSKP. In addition, CSKP strain (No. 20-18) was not grouped into neither CSKP nor CRKP groups.

Supplementary Table 1 The minimal inhibitory concentrations (MIC) breakpoint standards of the Clinical and Laboratory Standards Institute (CLSI) Subcommittee on Antimicrobial Susceptibility Testing (M100-S30) for CRKP strains.

**Antibiotics**	**CSLI Standards**	**CRKP**							
		**16-2**	**16-47**	**17-12**	**24-17**	**24-03**	**26-272**	**29-71**	**29-95**
Doxycycline	S:≤4, I:=8, R:≥16	MIC=8	MIC=8	MIC≥16	MIC≥16	MIC=8	MIC≥16	MIC≥16	MIC≥16
Cefuroxime Axetil	S:≤4, I:8-16, R:≥32	MIC≥64	MIC≥64	MIC≥64	MIC≥64	MIC≥64	MIC≥64	MIC≥64	MIC≥64
Amoxicillin-clavulanic acid	S;≤8/4, R:≥32/16	MIC≥32/16	MIC≥32/16	MIC≥32/16	MIC≥32/16	MIC≥32/16	MIC≥32/16	MIC≥32/16	MIC≥32/16
Minocycline	S≤4, I=8, R:≥16	MIC=16	MIC=16	MIC≥16	MIC=16	MIC=16	MIC≥16	MIC≥16	MIC≥16
Cephalothin	S:≤8, R:≥2	MIC≥64	MIC≥64	MIC≥64	MIC≥64	MIC≥64	MIC≥64	MIC≥64	MIC≥64
Amikacin	S:≤16, I;=32, R:≥64	MIC≥64	MIC≤2	MIC≤2	MIC=8	MIC≥64	MIC≤2	MIC≥64	MIC≥64
Cefepime	S:≤2, R:≥16	MIC≥32	MIC≥32	MIC≥32	MIC≥32	MIC≥32	MIC≥32	MIC≥32	MIC≥32
Cefotetan	S:≤16, I;=32, R:≥64	MIC=16	MIC=32	MIC≥64	MIC≥64	MIC=32	MIC=32	MIC≥64	MIC≥64
Cefotaxime	S:≤1, I:=2, R:≥4	MIC≥64	MIC≥64	MIC≥64	MIC≥64	MIC≥64	MIC≥64	MIC≥64	MIC≥64
Cefpodoxime	S:≤2, I:=4, R:≥8	MIC≥8	MIC≥8	MIC≥8	MIC≥8	MIC≥8	MIC≥8	MIC≥8	MIC≥8
Ceftazidime	S:≤4, I:=8, R:≥16	MIC≥64	MIC≥64	MIC≥64	MIC≥64	MIC≥64	MIC≥64	MIC≥64	MIC≥64
Ceftizoxime	S:≤1, I:=2, R:≥4	MIC=16	MIC=16	MIC=16	MIC=32	MIC=32	MIC=16	MIC=16	MIC=16
Cefuroxime	S:≤8, I:=16, R:≥32	MIC≥64	MIC≥64	MIC≥64	MIC≥64	MIC≥64	MIC≥64	MIC≥64	MIC≥64
Ciprofloxacin	S:≤0.25, I:=0.5, R:≥1	MIC≥4	MIC≥4	MIC≥4	MIC≥4	MIC≥4	MIC≥4	MIC≥4	MIC≥4
Imipenem	S:≤1, I:=2, R:≥4	MIC=2	MIC≥16	MIC≥16	MIC≥16	MIC≥16	MIC≥16	MIC≥16	MIC≥16
Levofloxacin	S:≤0.5, I:=1, R:≥2	MIC≥8	MIC≥8	MIC≥8	MIC≥8	MIC≥8	MIC≥8	MIC≥8	MIC≥8
Meropenem	S:≤1, I:=2, R:≥4	MIC≥16	MIC≥16	MIC≥16	MIC≥16	MIC≥16	MIC≥16	MIC≥16	MIC≥16
Moxifloxacin	S:≤0.05, R:≥2	MIC≥8	MIC≥8	MIC≥8	MIC≥8	MIC≥8	MIC≥8	MIC≥8	-
Nadine	S:≤16, R:≥32	MIC≥32	MIC≥32	MIC≥32	MIC≥32	MIC≥32	MIC≥32	MIC≥32	-
Norfloxacin	S:≤4, I:=8, R:≥16	MIC≥16	MIC≥16	MIC≥16	MIC≥16	MIC≥16	MIC≥16	MIC≥16	-
Piperacillin	S:≤16, I:=32-64, R:≥128	MIC≥128	MIC≥128	MIC≥128	MIC≥128	MIC≥128	MIC≥128	MIC≥128	-
Piperacillin-tazobactam	S:≤16/4, I:=32/4-64/4, R:≥128/4	MIC≥128/4	MIC≥128/4	MIC≥128/4	MIC≥128/4	MIC≥128/4	MIC≥128/4	MIC≥128/4	MIC≥128/4

tetracycline	S:≤4, I:=8, R:≥16	MIC=8	MIC=8	MIC≥16	MIC≥16	MIC=8	MIC=8	MIC≥16	-
Ticarcillin	S:≤16, I:=32-64, R:≥128	MIC≥128	MIC≥128	MIC≥128	MIC≥128	MIC≥128	MIC≥128	MIC≥128	MIC≥128
Ticarcillin-clavulanic acid	S:≤16/2, I:=32/2-64/2, R:≥128/2	MIC≥128/2	MIC≥128/2	MIC≥128/2	MIC≥128/2	MIC≥128/2	MIC≥128/2	MIC≥128/2	MIC≥128/2
Tobramycin	S:≤4, I:=8, R:≥16	MIC≥16	MIC=8	MIC≤1	MIC≥16	MIC≥16	MIC=8	MIC≥16	MIC≥16
Compound trimethoprim	S:≤2/38, R:≥4/76	MIC≥16/304	MIC=4/76	MIC≤1/19	MIC≥16/304	MIC≥16/304	MIC=4/76	MIC≤1/19	MIC≥16/304
Aztreonam	S:≤4, I:=8, R:≥16	MIC≥64	MIC≥64	MIC≥64	MIC≥64	MIC≥64	MIC≥64	MIC≥64	MIC≥64
Cefoperazone/Sulbactam	S:≤16/8, I:=32/16, R:≥64/32	MIC≥64/32	MIC≥64/32	MIC≥64/32	MIC≥64/32	MIC≥64/32	MIC≥64/32	MIC≥64/32	MIC≥64/32
Polymyxin	S≤2 、R≥4	MIC≤0.5	MIC≤0.5	MIC≤0.5	MIC≤0.5	MIC≤0.5	MIC≤0.5	MIC≤0.5	MIC≤0.5
Tigecycline	S≤0.5 、R≥8	MIC=2	-	-	-	-	-	-	-
Donipenem	S:≤1, I:=2, R:≥4	MIC≥8	MIC≥8	MIC≥8	MIC≥8	MIC≥8	MIC≥8	MIC≥8	-
Ceftazidime/Avibactam	S:≥21, R:≤20 (K-B Method)*	K-B=28	K-B=25	K-B=27	K-B=27	K-B=19	K-B=26	K-B=24	K-B=8

*K-B Method: Kirby-Bauer test, also known as the disk-diffusion method.

Supplementary Table 2 The minimal inhibitory concentrations (MIC) breakpoint standards of the Clinical and Laboratory Standards Institute (CLSI) Subcommittee on Antimicrobial Susceptibility Testing (M100-S30) for CSKP strains.

**Antibiotics**	**CSLI Standards**	**CSKP**						
		**16-14**	**16-9**	**18-2**	**18-28**	**18-88**	**20-18**	**30-45**
Doxycycline	S:≤4，I:=8,R:≥16	MIC=2	MIC=1	MIC≥16	MIC=2	MIC=8	MIC=8	MIC≥16
Cefuroxime Axetil	S:≤4，I:8-16,R:≥32	MIC=2	MIC=4	MIC≥64	MIC=4	MIC≥64	MIC≥64	MIC≥64
Amoxicillin-clavulanic acid	S;≤8/4，R:≥32/16	MIC≤2/1	MIC≤2/1	MIC=8/4	MIC=4/2	MIC=16/8	MIC=16/8	MIC=16/8
Minocycline	S≤4,I=8,R:≥16	MIC=2	MIC=2	MIC≥16	MIC=4	MIC≥16	MIC≥16	MIC≥16
Cephalothin	S:≤8，R:≥2	MIC≤2	MIC≤2	MIC≥64	MIC=4	MIC≥64	MIC≥64	MIC≥64
Amikacin	S:≤16，I;=32,R：≥64	MIC≤2	MIC≤2	MIC≤2	MIC≤2	MIC≥64	MIC≥64	MIC≤2
Cefepime	S:≤2,R：≥16	MIC≤0.12	MIC≤0.12	MIC=2	MIC≤0.12	MIC≥32	MIC≥32	MIC≥32
Cefotetan	S:≤16，I;=32,R：≥64	MIC≤4	MIC≤4	MIC≤4	MIC≤4	MIC=16	MIC=16	MIC≤4
Cefotaxime	S:≤1，I:=2,R:≥4	MIC≤1	MIC≤1	MIC≥64	MIC≤1	MIC≥64	MIC≥64	MIC≥64
Cefpodoxime	S:≤2，I:=4,R:≥8	MIC≤0.25	MIC≤0.25	MIC≥8	MIC≤0.25	MIC≥8	MIC≥8	MIC≥8
Ceftazidime	S:≤4，I:=8,R:≥16	MIC=4	MIC≤0.12	MIC=0.5	MIC=0.25	MIC≥64	MIC≥64	MIC=16
Ceftizoxime	S:≤1，I:=2,R:≥4	MIC≤1	MIC≤1	MIC≤1	MIC≤1	MIC≥64	MIC≥64	MIC≤1
Cefuroxime	S:≤8，I:=16,R:≥32	MIC=2	MIC=4	MIC≥64	MIC=4	MIC≥64	MIC≥64	MIC≥64
Ciprofloxacin	S:≤0.25，I:=0.5,R:≥1	MIC≤0.25	MIC≤0.25	MIC=1	MIC≤0.25	MIC≥4	MIC≥4	MIC=1
Imipenem	S:≤1，I:=2,R:≥4	MIC≤0.25	MIC=0.5	MIC≤0.25	MIC≤0.25	MIC≤0.25	MIC≤0.25	MIC≤0.25
Levofloxacin	S:≤0.5，I:=1,R:≥2	MIC≤ 0.12	MIC≤0.12	MIC=1	MIC≤0.12	MIC≥8	MIC≥8	MIC=1
Meropenem	S:⩽1，I:=2,R:⩾4	MIC⩽0.25	MIC⩽0.25	MIC⩽0.25	MIC⩽0.25	MIC=0.5	MIC=0.5	MIC⩽0.25
Moxifloxacin	S:≤0.05，R:≥2	MIC⩽0.25	MIC⩽0.25	MIC=2	MIC⩽0.25	MIC⩾8	MIC⩾8	MIC=4
Nadine	S:⩽16，R:⩾32	MIC=4	MIC=4	MIC=16	MIC=8	MIC⩾32	MIC⩾32	MIC=16
Norfloxacin	S:≤4，I:=8,R:≥16	MIC≤0.5	MIC≤0.5	MIC=2	MIC≤0.5	MIC≥16	MIC≥16	MIC=2
Piperacillin	S:≤16，I:=32-64,R:≥128	MIC=64	MIC=16	MIC≥128	MIC=32	MIC≥128	MIC≥128	MIC≥128
Piperacillin-tazobactam	S:≤16/4，I:=32/4-64/4,R:≥128/4	MIC≤4/1	MIC≤4/1	MIC≤4/1	MIC=8/2	MIC≥128/4	MIC≥128/4	MIC=8/2

tetracycline	S:≤4，I:=8,R:≥16	MIC≤1	MIC≤1	MIC≥16	MIC≤1	MIC=8	MIC=8	MIC≥16
Ticarcillin	S:≤16，I:=32-64,R:≥128	MIC≥128	MIC≥128	MIC≥128	MIC≥128	MIC≥128	MIC≥128	MIC≥128
Ticarcillin-clavulanic acid	S:≤16/2，I:=32/2-64/2,R:≥128/2	MIC≤8/1	MIC≤8/1	MIC=16/2	MIC≤8/1	MIC≥128/2	MIC≥128/2	MIC=64/2
Tobramycin	S:≤4，I:=8,R:≥16	MIC≤1	MIC≤1	MIC=2	MIC≤1	MIC≥16	MIC≥16	MIC≤1
Compound trimethoprim	S:≤2/38,R:≥4/76	MIC≤1/19	MIC≤1/19	MIC≤1/19	MIC≤1/19	MIC≥16/304	MIC≥16/304	MIC≤1/19
Aztreonam	S:≤4，I:=8,R:≥16	MIC≤1	MIC≤1	MIC≤1	MIC≤1	MIC≥64	MIC≥64	MIC=16
Cefoperazone/Sulbactam	S:≤16/8，I:=32/16,R:≥64/32	MIC≤8/4	MIC≤8/4	MIC≤8/4	MIC≤8/4	MIC≥64/32	MIC≥64/32	MIC≤16/8
Polymyxin	S≤2 、R≥4	MIC≤0.5	MIC≤0.5	MIC≤0.5	MIC≤0.5	MIC≤0.5	MIC≤0.5	MIC≤0.5
Tigecycline	S≤0.5 、R≥8	MIC≤0.5	MIC≤0.5	MIC=1	MIC≤0.5	MIC=2	MIC=2	MIC=2
Donipenem	S:≤1，I:=2,R:≥4	MIC=0.25	MIC=0.12	MIC≤0.12	MIC≤0.12	MIC=0.5	MIC=0.5	MIC≤0.12

Supplementary Table 3 The baseline information of *K. pneumoniae* infected patients and the total number of SERS spectra for each of *K. pneumoniae* strains.

Strains	No. of Strains	Sample ID	Clinical Sample	No. of SERS Spectra	No. of Total Spectra
CSKP	7	16-14	Sputum	50	280
		16-9	Sputum	30	
		18-2	Sputum	30	
		18-28	Sputum	30	
		18-88*	Sputum	30	
		20-18*	Sputum	50	
		30-45	Sputum	60	
CRKP	8	16-2	Sputum	20	280
		16-47	Sputum	30	
		17-12	Sputum	30	
		24-03	Sputum	20	
		24-17	Sputum	30	
		26-272	Blood	30	
		29-71	Sputum	60	
		29-95	Sputum	60	

*The two *K. pneumoniae* strains were independently isolated from the same patient.
